# The prevalence of tuberculosis, malaria and soil-transmitted helminth infection in minority indigenous people of Southeast Asia and the Western Pacific: protocol for a systematic review and meta-analysis

**DOI:** 10.1186/s13643-021-01753-y

**Published:** 2021-07-10

**Authors:** Beth Gilmour, Kefyalew Addis Alene, Naomi E. Clarke, Archie C. A. Clements

**Affiliations:** 1 Faculty of Health Sciences, Curtin University, Western Australia, Australia; 2 Telethon Kids Institute, West Perth, WA Australia; 3 Research School of Population Health, Australian National University, Canberra, ACT Australia

**Keywords:** Tuberculosis, TB, Malaria, Soil-transmitted helminth, STH, Indigenous, Minority, Southeast Asia, Western Pacific, Systematic review

## Abstract

**Background:**

Infectious diseases such as tuberculosis (TB), malaria and soil-transmitted helminthiasis continue to impose a significant global health burden and socio-economic impact. Globally, minority indigenous people are disproportionately affected by poverty and are shown to experience a disparate burden of disease and poorer health outcomes than the comparative majority population. Despite these inequalities, countries rarely systematically compile epidemiological data disaggregated by ethnicity to enable the extent of the differential to be quantified.

**Methods:**

The systematic review will be reported in accordance with The Preferred Reporting Items for Systematic Review and Meta- Analyses (PRISMA) guidelines. Systematic searches will be conducted in EMBASE, Medline, Scopus and Web of Science for studies reporting data which enable the prevalence of TB, malaria, and/or soil-transmitted helminth (STH) infections amongst minority indigenous populations within the Southeast Asia Region (SEAR) and Western Pacific Region (WPR) to be calculated.

Where studies provide data on disease prevalence for both minority indigenous and other populations within the same study, a comparative analysis will be undertaken. In addition to a narrative synthesis, where sufficient data are available, a random-effects meta-analysis will be conducted to obtain a pooled estimate value for each disease/infection by country and mortality stratum.

Heterogeneity between studies will be examined using the Cochran’s Q test and quantitatively measured by the index of heterogeneity squared (I^2^) statistics. The methodological quality of the included studies will be assessed using a modified Newcastle-Ottawa Scale.

**Discussion:**

This systematic review aims to analyse the available data on the prevalence of TB, malaria and STH infections within minority indigenous populations of the SEAR and WPR.

**Registration:**

Open Science Framework registration: osf.io/m6sqc

**Supplementary Information:**

The online version contains supplementary material available at 10.1186/s13643-021-01753-y.

## Background

Despite impacting human health since ancient times [1–3], tuberculosis (TB), malaria, and soil-transmitted helminth (STH) infections continue to create a significant social and economic burden.

TB, an airborne bacterial disease caused by the bacterium *Mycobacterium tuberculosis* ranks in the top ten causes of death worldwide, killing more than 1.5 million people in 2018 [4]. TB is second to Coronavirus disease 2019 (COVID-19) as a leading cause of death due to a single infectious agent [5].

The protozoan parasite *Plasmodium* spp., transmitted via the female *Anopheline* mosquito vector, is responsible for causing malaria. In 2018, malaria is estimated to have caused 228 million cases of disease and 405,000 deaths worldwide [6].

STH infections are a neglected tropical disease (NTD) caused by parasitic nematode worms, including *Ascaris lumbricoides* (roundworm), *Trichuris trichiura* (whipworm), and *Necator americanus* and *Ancylostoma* spp. (hookworm). Together, these parasites are thought to infect more than 1.5 billion people [7], a figure which equates to 19% of the world’s population. Although currently excluded from STH statistics, *Strongyloides stercoralis* is another pathogenic nematode of significance to human health.

Despite rarely causing mortality, STH infections are of major significance with respect to their burden of morbidity [8] and they are the most prevalent of the NTDs as defined by the World Health Organization (WHO) [9].

In 2016, 51.6 million disability adjusted life years (DALYs) were lost due to TB, 37.3 million DALYs were lost due to malaria and 3.4 million DALYs were lost due to STH infections globally [10]. These three diseases also have a substantial impact on the global economy. TB-related mortality was estimated to cause the loss of 616 billion USD between 2000 and 2015 and is projected to lead to a further loss of 984 billion USD between 2015 and 2030 [11]. Countries where severe malaria (malaria index > 0.5) is endemic are estimated to experience a 1.3% lower economic growth rate per annum [12]. The economic impact of STH infections is difficult to quantify, but mathematical modelling estimates the impact of hookworm infection to cost $2.5 to $138.9 billion per annum [13].

The organisms responsible for TB, malaria and STH infections are endemic in the tropics and are more prevalent amongst populations living in poverty [7, 14–17]. Globally, minority indigenous people are shown to be disproportionately affected by poverty, with their representation amongst the poor reaching 60–70% in some regions [18]. Minority indigenous people experience a disproportionate burden of disease and poorer health outcomes than their majority population counterparts [19–21].

In 2015, all member states of the United Nations endorsed the 2030 Agenda for Sustainable Development [22]. This is an ambitious agenda that calls on all countries to end poverty whilst achieving social, economic and environmental sustainability in an equitable manner [22]. A commitment of the Agenda is that “no one will be left behind” and that endeavours will be made to “reach the furthest behind first” [22].

The effective transformation of the Agenda goals into realistic interventions requires an accurate understanding of target populations and their relative disease burden [23]. At present, data and current indicators are rarely disaggregated to facilitate the identification of vulnerable groups [23].

Although studies have been undertaken on the prevalence of infectious diseases within individual indigenous groups, disease burden is not well understood within the context of minority indigenous people as a collective. Data on these vulnerable groups are crucial in facilitating achievement of the 2030 Sustainable Development Goals and enabling industrialized nations to narrow the health gap between their minority indigenous and majority populations.

A systematic review of disease prevalence in minority indigenous populations will provide a baseline and identify data gaps for this vulnerable population group as a collective.

This paper describes the protocol for a systematic review to determine the prevalence of TB, malaria, and STH infections among minority indigenous people of the WHO Southeast-Asia Region (SEAR) and Western Pacific Region (WPR). TB, malaria, and STH infections have been chosen as they are of major global health significance, and they have social determinants (such as poverty and health service inaccessibility) that make minority indigenous people particularly vulnerable to infection. The SEAR and WPR have been chosen to capture a significant proportion of the world’s indigenous people [24] whilst also providing an opportunity to compare data across countries with differing levels of socio-economic development.

## Methods/design

This protocol is reported in accordance with Preferred Reporting Items for Systematic Review and Meta-Analysis Protocols (PRISMA-P) guidelines [25], the checklist for which is detailed in Additional file 1. If there is a need to amend this protocol, the date of each amendment and the reason for the change will be described.

### Search strategy

A systematic search for epidemiological studies will be conducted in four biomedical databases: EMBASE (Ovid), Medline (Ovid), Scopus, and Web of Science. The search strategy has been developed with the help of a professional librarian and will be undertaken without restriction on the year of publication. Grey literature and regional databases will be included in the search and reference lists from relevant studies hand-searched. Forward and backward citation searching will be undertaken using Google Scholar to identify related articles. Authors of relevant papers will be contacted when there is a need for additional information.

The WHO Global Burden of Disease (GBD) regional classification system [26] will be used to define the countries within the SEAR and WPR. Singapore will be excluded as it does not have any minority indigenous people according to the definitions utilized by this review.

Although there is no universally accepted definition of ‘indigenous status,’ the United Nations (UN) and the International Labour Organization (ILO) Indigenous and Tribal People Convention (#169) utilise a number of attributes to define indigenous people [27, 28]. For the purposes of this review, the UN attributes will be included, and indigenous minorities will be defined as population groups who meet each of the following criteria:

- Descendants of the original or earliest known inhabitants of an area; people who have historical continuity with pre-invasion and pre-colonial societies [28–30]
- Distinct societies with languages, culture, customs, and social and political frameworks which vary significantly from those of the dominant population [18, 28–31]
- Groups of people with strong cultural ties and dependence upon the environment and its resources for their survival [21, 28, 29, 31]
- People self-identifying as indigenous [28]
- Groups who face relative disadvantage or discrimination in multiple areas of social existence—success, education, healthcare, employment [19, 28, 32]
- Numerically non-dominant groups in a country or area [28]

In addition to universal indigenous terms, those relevant to each country will be used as detailed in Additional file 1. Country-specific indigenous terms have been derived from the World Directory Listing of Minorities and Indigenous People [33], Native Planet–Indigenous Mapping [34], and the International Working Group on Indigenous Affairs [35].

The following search terms will be used to identify studies on TB, malaria, and STH infections: “soil transmitted helminth*” OR STH OR *Ascaris* OR *Trichuris* OR *Nectator* OR *Ancylostoma* OR hookworm* OR *Strongyloides* OR malaria* OR plasmodi* OR tuberculosis OR TB OR “*Mycobacterium tuberculosis*”. The Plasmodium and helminth species that will be included within the review are detailed in the inclusion criteria. An example search strategy for Indonesia is detailed in Additional file 1.

### Study selection

All articles identified from the systematic search will be uploaded into Endnote X9 (Clarivate Analytics) and duplicate articles removed. Two researchers (BG and KAA) will independently screen the titles and abstracts of the studies on Rayyan QCRI [36] and will then review the full text against the eligibility criteria. Any disagreements will be resolved through discussion and, in the event consensus cannot be achieved, agreement will be reached following discussions with a third author (ACAC).

### Inclusion criteria

Studies are required to meet each of the following inclusion criteria:

- Studies that relate to human infection: for malaria, studies on *Plasmodium vivax*, *Plasmodium falciparum*, *Plasmodium ovale,* and *Plasmodium malariae* which undergo human-to-human transmission and the zoonotic species *Plasmodium knowlesi*; for STH infections, studies on *A. lumbricoides* (roundworms), *T. trichiura* (whipworms), *N. americanus* and *Ancylostoma duodenale* (hookworms), *Strongyloides stercoralis* (threadworms), and the zoonotic hookworm species *Ancylostoma ceylanicum, Ancylostoma caninum,* and *Ancylostoma braziliense*
- Studies including minority indigenous populations
- Studies that report sufficient data to facilitate the calculation of TB, malaria, or STH prevalence
- Studies conducted within the SEAR or WPR as defined by the WHO regional classification system [26]
- Cross-sectional studies/ representative surveys
- Where studies undertake analyses pre and post intervention regimes, only pre-intervention baseline data will be recorded

### Exclusion criteria

Studies will be excluded if they meet any of the following criteria:

- Case studies
- Case series with < 10 people
- Scientific correspondence, poster, and conference abstracts
- Systematic or literature reviews
- Due to resource constraints, articles not published in English will be excluded
- Papers where minority indigenous people comprise less than 90% of the group stated to be an indigenous minority population for the purpose of calculating prevalence in the indigenous minority group
- Studies on latent TB; diagnostic methods must be able to confirm active disease (i.e., studies utilizing Mantoux testing as the sole diagnostic will be excluded)

### Data extraction

Data from the included studies will be independently extracted in a Microsoft Excel (version 2014) spreadsheet by BG and KAA. The data extraction spreadsheet will be piloted on five papers and then refined, if needed. Corresponding authors will be contacted by e-mail if relevant information is missing or unclear. If clarifications are not received within 4 weeks, the study will be excluded.

Where available, the following data will be extracted from each eligible publication: first author, year of publication, year of study, geographic location of study population (country, region), sample size, demographic factors (age group and sex), study design, bacteria/parasite species, number of people within sample population who are infected, diagnostic method utilised, number of samples taken and analyzed per participant, study population (minority indigenous/other), name of minority indigenous group, co-infection (name of infectious agent), and number of participants co-infected with multiple infectious agents.

Where studies undertake a comparison between minority indigenous and other population groups, data will be extracted for both groups to facilitate a comparison. A data extraction tool is provided in Additional file 1.

### Quality and bias assessment

The methodological quality of the included studies will be assessed by two investigators (BG and KAA) using a modified version of the Newcastle-Ottawa Quality Assessment Scale [37] as detailed in Additional file 1. The quality assessment tool will be piloted on 10 randomly selected papers to increase agreement between the two reviewers, and any subsequent differences will be resolved through discussion with a third reviewer (ACAC). The QA tool has scores ranging from 0 to 9; scores between 1 and 4 will be defined as low quality, scores between 5 and 7 will be defined as medium quality, and scores between 8 and 9 will be defined as high quality. Sensitivity analyses will be performed to assess the impacts of methodological quality on the results of the review.

Funnel plots will be used to detect potential publication bias and small study effects. Egger’s method will be used to assess asymmetry, with a P value < 0.05 considered to indicate statistically significant publication bias [38].

### Quantitative analysis

The primary outcomes are the prevalence of TB, malaria, and STH infection among minority indigenous populations within the SEAR and WPR and across different mortality strata as defined by the WHO [26].

A random-effects meta-analysis will be used to obtain a pooled estimate value for each of the outcomes of interest. Where sufficient studies are available (three or more studies), subgroup analysis will be performed to assess the effects of each study characteristic on the primary outcomes of the study. A comparison will be made between minority indigenous and other population groups if sufficient data are available from studies that compare these groups directly. Heterogeneity between studies will be examined using the Cochran’s Q test and quantitatively measured by the index of heterogeneity squared (I^2^) statistics with 95% confidence intervals (CI) [39]. Heterogeneity between studies will be considered low, moderate, and high when I^2^ values are below 25%, between 25% and 75%, and above 75%, respectively [39]. When there is evidence of significant heterogeneity, the sources of heterogeneity will be explored through meta-regression using study characteristics (e.g., country, mortality strata, diagnostic method) as covariates. The analysis will be conducted in Stata/MP version 18 (StataCorp, College Station, TX, USA).

## Discussion

To address the issues of poverty, inequality, and the impact of infectious diseases such as TB, malaria, and soil-transmitted helminthiasis, several global goals and strategies have been endorsed. These include the 2030 Sustainable Development Agenda [22], the WHO 2016–2035 End TB Strategy [40], the WHO Global Technical Strategy for Malaria 2016–2030 [41], and the WHO 2030 targets for STH control programs [42].

Due to poverty, increased exposure to proximal determinants of disease, and living in remote and isolated locations, minority indigenous people have been shown to experience a disparate burden of TB, malaria, and STH infections [43–47].

These health inequalities are significant in all societies because, although minority indigenous people living in industrialized countries have a lower burden of disease relative to those living in developing countries, the differential in disease burden between indigenous and majority populations has been shown to be greater in industrialized nations [44].

If the WHO targets and the 2030 Sustainable Development Agenda goals are to be accomplished, the prevalence of infectious diseases amongst vulnerable groups needs to be quantified. The WHO Constitution defines health as “a state of complete physical, mental, and social well-being and not merely the absence of disease or infirmity” [48]. This definition highlights a holistic approach which more closely aligns with the harmonious lifestyle fundamental to indigenous culture [49]. To be successful, health systems need to respect indigenous culture [50] and embrace its positive attributes [51]. The findings of this systematic review will identify data gaps and provide information on the prevalence of disease burden which can be used to inform strengths based and community-led intervention.

## Supplementary Information

**Additional file 1 Appendix 1:** PRISMA-P 2015 Checklist. **Appendix 2:** Search Criteria. **Appendix 3:** Example search strategy for Indonesia. **Appendix 4:** Data extraction tool. **.Appendix 5:** Quality and bias assessment.

## Data Availability

All the required information is available in the manuscript and supporting documents.

## Abbreviations

CI: Confidence Interval
DALY: Disability adjusted life year
GBD: Global Burden of Disease
I^2^: Index of heterogeneity squared
ILO: International Labour Organization
NTD: Neglected tropical disease
PRISMA-P: Preferred Reporting Items for Systematic Review and Meta-Analysis Protocols
SEAR: Southeast Asia Region
STH: Soil-transmitted helminth
TB: Tuberculosis
UN: United Nations
WHO: World Health Organization
WPR: Western Pacific Region
