# Supplementary material for: The prevalence of tuberculosis, malaria and soil-transmitted helminth infection in minority indigenous people of Southeast Asia and the Western Pacific: protocol for a systematic review and meta-analysis
Source: Syst Rev. 2021 Jul 10;10:203. doi: 10.1186/s13643-021-01753-y (PMC8271287; doi:10.1186/s13643-021-01753-y)
Supplement: Supplementary file 1 — Additional file 1 Appendix 1: PRISMA-P 2015 Checklist. Appendix 2: Search Criteria. Appendix 3: Example search strategy for Indonesia. Appendix 4: Data extraction tool. .Appendix 5: Quality and bias assessment. [file 13643_2021_1753_MOESM1_ESM.docx]

**Supplementary information**

**Appendix 1:** PRISMA-P 2015 Checklist *

| **Section/topic** | **#** | **Checklist item** | **Information reported** | | **Line number (s)** |
| --- | --- | --- | --- | --- | --- |
|  |  |  | **Yes** | **No** |  |
| **ADMINISTRATIVE INFORMATION** | | | | | |
| **Title** | | | | | |
| Identification | 1a | Identify the report as a protocol of a systematic review |  |  | 2-3, 113, 124-126 |
| Update | 1b | If the protocol is for an update of a previous systematic review, identify as such |  |  |  |
| **Registration** | 2 | If registered, provide the name of the registry (e.g., PROSPERO) and registration number in the Abstract |  |  | 52 |
| **Authors** | | | | | |
| Contact | 3a | Provide name, institutional affiliation, and e-mail address of all protocol authors; provide physical mailing address of corresponding author |  |  | 5-20 |
| Contributions | 3b | Describe contributions of protocol authors and identify the guarantor of the review |  |  | 311-313 |
| **Amendments** | 4 | If the protocol represents an amendment of a previously completed or published protocol, identify as such and list changes; otherwise, state plan for documenting important protocol amendments |  |  | 126-127 |
| **Support** | | | | | |
| Sources | 5a | Indicate sources of financial or other support for the review |  |  | 306-308 |
| Sponsor | 5b | Provide name for the review funder and/or sponsor |  |  |  |
| Role of sponsor/funder | 5c | Describe roles of funder(s), sponsor(s), and/or institution(s), if any, in developing the protocol |  |  | 309 |
| **INTRODUCTION** | | | | | |
| **Rationale** | 6 | Describe the rationale for the review in the context of what is already known |  |  | 25-31, 58-121 |
| **Objectives** | 7 | Provide an explicit statement of the question(s) the review will address with reference to participants, interventions, comparators, and outcomes (PICO) |  |  | 241-248 |
| **METHODS** | | | | | |
| **Eligibility criteria** | 8 | Specify the study characteristics (e.g., PICO, study design, setting, time frame) and report characteristics (e.g., years considered, language, publication status) to be used as criteria for eligibility for the review |  |  | 128-167, 177-206 |
| **Information sources** | 9 | Describe all intended information sources (e.g., electronic databases, contact with study authors, trial registers, or other grey literature sources) with planned dates of coverage |  |  | 128-136 |
| **Search strategy** | 10 | Present draft of search strategy to be used for at least one electronic database, including planned limits, such that it could be repeated |  |  | 505-575; 584-614 |
| ***STUDY RECORDS*** | | | | | |
| Data management | 11a | Describe the mechanism(s) that will be used to manage records and data throughout the review |  |  | 170-171, 209-211 |
| Selection process | 11b | State the process that will be used for selecting studies (e.g., two independent reviewers) through each phase of the review (i.e., screening, eligibility, and inclusion in meta-analysis) |  |  | 171-175, 209-210, 227-229 |
| Data collection process | 11c | Describe planned method of extracting data from reports (e.g., piloting forms, done independently, in duplicate), any processes for obtaining and confirming data from investigators |  |  | 210-213 , 229-231, 623-649 |
| **Data items** | 12 | List and define all variables for which data will be sought (e.g., PICO items, funding sources), any pre-planned data assumptions and simplifications |  |  | 214-221, 623-649 |
| **Outcomes and prioritization** | 13 | List and define all outcomes for which data will be sought, including prioritization of main and additional outcomes, with rationale |  |  | 36-43, 241-248 |
| **Risk of bias in individual studies** | 14 | Describe anticipated methods for assessing risk of bias of individual studies, including whether this will be done at the outcome or study level, or both; state how this information will be used in data synthesis |  |  | 226-238 |
| ***DATA*** | | | | | |
| **Synthesis** | 15a | Describe criteria under which study data will be quantitatively synthesized |  |  | 41-43, 244-251 |
|  | 15b | If data are appropriate for quantitative synthesis, describe planned summary measures, methods of handling data, and methods of combining data from studies, including any planned exploration of consistency (e.g., *I* ^2^, Kendall’s tau) |  |  | 248-255 |
|  | 15c | Describe any proposed additional analyses (e.g., sensitivity or subgroup analyses, meta-regression) |  |  | 255-258 |
|  | 15d | If quantitative synthesis is not appropriate, describe the type of summary planned |  |  |  |
| **Meta-bias(es)** | 16 | Specify any planned assessment of meta-bias(es) (e.g., publication bias across studies, selective reporting within studies) |  |  | 226-238 |
| **Confidence in cumulative evidence** | 17 | Describe how the strength of the body of evidence will be assessed (e.g., GRADE) |  |  | 246-251 |

The above checklist has been downloaded from BCM Systematic Reviews^1^ and has been adapted from the work undertaken by Moher et al, 2015 ^2^ with the rationale for the adaptation detailed in recommendations to prospective authors ^3^.

**Appendix 2:** Search Criteria

1. **Countries**

Countries comprising the SEAR and WPR are defined based on the WHO Global Burden of Disease (GBD) regional classification system ^4^.

| SEAR Category B ^#^ | SEAR Category D ^#^ |
| --- | --- |
| Indonesia | Bangladesh |
| Sri Lanka | Bhutan |
| Thailand | Korea, Democratic People’s Republic of |
| Timor-Leste | India |
|  | Maldives |
|  | Myanmar |
|  | Nepal |

| WPR Category A ^#^ | WPR Category B ^#^ |
| --- | --- |
| Australia | Cambodia |
| Brunei | China |
| Japan | Cook Islands |
| New Zealand | Fiji |
| Singapore* | Kiribati |
|  | Korea, Republic of |
|  | Lao |
|  | Malaysia |
|  | Marshall Islands |
|  | Micronesia |
|  | Mongolia |
|  | Nauru |
|  | Niue |
|  | Palau |
|  | Papua New Guinea |
|  | Philippines |
|  | Samoa |
|  | Solomon Islands |
|  | Tonga |
|  | Tuvalu |
|  | Vanuatu |
|  | Vietnam |

* Singapore will be excluded as it does not have any minority indigenous people according to the definitions utilized by this review.

^#^ Countries are classified according to mortality strata ^4^ :

Category A: very low child, very low adult mortality

Category B: low child, low adult mortality

Category C: Low child, high adult mortality (there are no Category C countries within the SEAR and WPR)

Category D: high child, high adult mortality

Category E: High child, very high adult mortality (there are no Category E countries within the SEAR and WPR)

Mortality strata are based upon the quintiles of distribution for adult and child mortality across WHO member states using 1999 population estimates ^5^.

1. **Parasites/Bacteria**

The following search terms will be used to identify studies on TB, malaria, and STH infections: “soil transmitted helminth*” OR STH OR Ascaris OR Trichuris OR Nectator OR Ancylostoma OR hookworm* OR Strongyloides OR malaria* OR plasmodi* OR

tuberculosis OR TB OR “Mycobacterium tuberculosis”

1. **Indigenous Terms**

In addition to generic indigenous terms, those relevant to each country have been derived from the World Directory Listing of Minorities and Indigenous People ^6^; Native Planet- Indigenous Mapping ^7^ and International Working Group on Indigenous Affairs ^8^ and are detailed below:

| INDONESIA: SEAR B |
| --- |
| Indigenous OR aborigin* OR native OR first nation* OR “ethnic group” OR tribal OR tribe OR autochthonous OR “adat terpencil” OR Acehnese OR Achinese OR Atjeher OR “Orang Aceh” OR Acehnais OR Acehno OR Atjeh OR Atjehnese OR Achehnese OR Achenese OR Adabe OR Ataura OR Atauru OR Atauro OR Raklu-Un OR “Raklu Un” OR Adonara OR “Tusa Tadon” OR Waiwerang OR Vaiverang OR Sagu OR Alorese OR Ampanang OR Andio OR Masama OR Andio'o OR Imbao'o OR Aralle OR Tabulahan OR Asmat OR Asamat OR Asemer OR Asomat OR Bagusa OR “Batak Alas-Kluet” OR “Alas-Kluet Batak” OR “Batak Kluet-Alas” OR “Kluet-Alas Batak” OR “Alas Kluet” OR “Kluet Alas” OR Alas OR Kluet OR “Batak Angkola” OR “Orang Angkola” OR Anakola OR Angkola OR “Batak Dairi” OR Dairi OR “Dairi Batak” OR “Orang Batak Dairi” OR Pakpak OR “Pakpak Dairi” OR Sumut OR “Batak Karo” OR “Karo Batak” OR “Orang Batak Karo” OR Karonese OR “Batak Mandailing” OR “Mandailing Batak” OR Batta OR “Orang Mandailing” OR “Batak Simalungun” OR “Simalungun Batak” OR “Orang Batak Simalungun” OR Simelungun OR Simelungan OR Timur OR “Batak Toba” OR “Toba Batak” OR “Orang Batak Toba” OR “Silindung Batak” OR Bauzi OR Baudi OR Bauri OR Baudji OR Baudzi OR Damal OR Uhunduni OR Amung OR “Amung Kal” OR Amungme OR Amuy OR Enggipiloe OR Hamung OR Oehoendoeni OR Dani OR Gayo OR “Orang Gayo” OR Gayonese OR Ketengban OR Kupel OR Oktengban OR Kombai OR Komboy OR Kubu OR Djambi OR “Orang Darat” OR Mentawai OR Mentawei OR Mentawi OR Minangkabau OR Minang OR Padang OR “Orang Minangkabau” OR Moni OR Migani OR Djonggunu OR Jonggunu OR Moronene OR Maronene OR Nias OR Batu OR Nuaulu OR “Southern Nuaulu” OR “Northern Nuaulu” OR Rejang OR “Keme Tun Djang” OR “Orang Rejang” OR Djang OR “Tun Djang” OR “Redjang Empat Petulai” OR “Djang Lebong” OR “Djang Bele Tebo” OR “Djang Musai” OR “Djang Lai” OR “Djang Bekulau” OR “Djang Abeus” OR “Djang Aweus” OR “Bang Hadji” OR Semitul OR Sawang OR Selako OR “Selako Dayak” OR Selakau OR Salakau OR Salako OR Silakau OR Tamiang OR Malayu OR Wandamen OR Wandamen-Windesi OR Windesi OR Windessi OR Bintuni OR Bentuni OR Bentoeni OR Wamesa OR Wolio OR Buton OR Butonese OR Walio |

| “SRI LANKA” OR CEYLON: SEAR B |
| --- |
| Indigenous OR aborigin* OR native OR “first nation*” OR “ethnic group” OR tribal OR tribe OR autochthonous OR “Ceylon Tamils” OR “Jaffna Tamils” OR “Indian Tamils” or “Estate Tamils” OR “Sri Lankan Moors” OR Burghers OR “Sri Lankan Chetty” OR Bharatha OR Wanniyala-Aetto OR Veddhas OR Sinhalese OR Tamil OR Wanniya-laeto OR Vedda OR Veddha OR Veddah OR Wanniyala-Aetto |

| THAILAND: SEAR B |
| --- |
| Indigenous OR aborigin* OR native OR “first nation*” OR “ethnic group” OR tribal OR tribe OR autochthonous OR Akha OR Hmong OR Karen OR Lahu OR Lisu OR Mein OR Mon OR “Khmer Thai Isan” OR “Thai Lao” OR Khmer OR Kaw OR Bisu OR Mbi OR Mbisu OR Mibisu OR Misu OR “Hmong Daw” OR “White Meo” OR “White Hmong” OR “Hmong Njua” OR “Black Meo” OR “Blue Meo” OR H'tin OR T'in OR Ht'in OR Thin OR Tin OR Khatin OR Isan OR Lao OR Isaan OR Issan OR Esarn OR Karen S’gaw OR Khmu OR Khamu OR Kammu OR Kui OR Kuoy OR Kuy OR Suoy OR Suay OR Suai OR Lahu OR Musser OR Lisu OR Lisaw OR “lu Mien” OR Mien OR Yao OR “Yui Mien” OR Mani OR Manik OR Maniq OR Negrito OR Mannee OR Mlabri OR “Phi Tong Luang” OR Moken OR Salong OR Selung OR Salone OR “Sea Gypsy” OR Moklen OR “Chao Lay” OR Palaung OR “Silver Palaung” OR “Pale Palaung” OR Bulay OR Dlang OR Palay OR Palong OR Pulei OR Shwe OR Ta’ang OR “Tai Lue” OR “Dai Lue” OR “Urak Lawoi*” OR “Chao Lay” OR “Lumoh Lawoi” OR “Sea Gypsies” OR “Thai Mai” OR “Chao thale” OR “Chao khao” OR “Chon phao” OR “Chon phao mueang” |

| TIMOR*: SEAR B |
| --- |
| Indigenous OR aborigin* OR native OR “first nation*” OR “ethnic group” OR tribal OR tribe OR autochthonous OR “Tetum Prasa” OR Mambai OR Makasae OR “Tetum Terik” OR Baikenu OR Kemak OR Bunak OR Tokodede OR Fataluku OR Waima’a OR Galoli OR Naueti OR Idate OR Midiki OR Tentum OR Baikeno OR Makasai |

| BANGLADESH: SEAR D |
| --- |
| Indigenous OR aborigin* OR native OR “first nation*” OR “ethnic group” OR tribal OR tribe OR autochthonous OR Adivasis OR Jumma OR Chakmas OR Marma OR Tripura OR Mro OR Biharis OR Chakma OR Takam OR Chakama OR Tsakma OR Changma OR “Changma Vaj” OR “Changma Kodha” OR Chin OR Khumi OR Khumi OR Khami OR Kami OR Kumi OR Khweymi OR Khuni OR Darlong OR Dalong OR Zo OR Garo OR A'Chik OR Mande OR Mandi OR Lamdani OR Achchik OR Acchiks OR Achik OR Oraon OR Uraon OR Khurukh |

| BHUTAN: SEAR D |
| --- |
| Indigenous OR aborigin* OR native OR “first nation*” OR “ethnic group” OR tribal OR tribe OR autochthonous OR Lhotshampas OR Chali OR Dakpa OR Sagtengpa OR Brokpa OR Brokkat OR Layap OR Lepcha OR Rong OR Rongke OR Rongpa OR Lhop OR Doya OR Lhokpu OR Lhops OR Lhopu OR Lhotshampas OR Gurkhali OR Nepali OR Paharia OR “Southern Bhutanese” OR Monpa OR Menba OR Moinba OR Monba OR Menpa OR Mongba OR Ngalop OR Bhote OR Sharchop OR Schachop OR Bhotia OR “Central Monba” OR “Cuona Monba” OR Memba OR Sarchapkkha OR “Southern Moonba” OR Tshalingpa OR “Bhotia Eastern” OR “Cona Monba” OR “Eastern Bhutanese” OR Mompa OR Sangla OR Sharchagpakha OR Tsangla |

| “DEMOCRATIC PEOPLE’S REPUBLIC OF KOREA”: SEAR D |
| --- |
| Indigenous OR aborigin* OR native OR “first nation*” OR “ethnic group” OR tribal OR tribe OR autochthonous |

| INDIA: SEAR D |
| --- |
| Indigenous OR aborigin* OR native OR “first nation*” OR “ethnic group” OR tribal OR tribe OR autochthonous OR Dalits OR Nagas OR Adivasis OR Adaman* OR Onges OR Jarawa OR Sentinelese OR “Adi Padam” OR Padam OR Miri OR Abor OR Arbor OR Abor-Miri OR Aimol OR Angami OR “Southern Angami” OR Japfuphiki OR “Western Angami” OR Jotsoma OR Khonoma OR Mezoma OR Chakhro OR “Northern Angami” OR Ao OR Awan OR “QuTB Shahi Awan” OR Badaga OR Badag OR Badagux OR Badugu OR Vadagu OR Baiga OR Bhumia OR Bhuiya OR Narotia OR Binjwar OR Bharotia OR Raibhaina OR Kathbhaina OR Kondwan OR Gonwaina OR Bangni OR Dafla OR Nishi OR “Nishi Bangni” OR Banjara OR Vanzara OR Lambadi OR Sugali OR Ghor OR Bharia OR Bhar OR Bharat OR Bhumia OR Bhumiya OR Paliha OR “Bhuinha Bhumia” OR Bhumiya OR Pando OR Bhil OR Bhilbari OR Bhilboli OR Bhilla OR Bhili OR Bhilodi OR Vil OR Bhagoria OR Lengotia OR Birhor OR Bihor OR Birhar OR Birhore OR Mankidi OR Mankidia OR Bishnoi OR Marwadi OR Vishnoi OR Bodo OR Boro OR Bodi OR Bara OR Boroni OR Mechi OR Meche OR Mech OR Meci OR Kachari OR Bondo OR “Bondo Poraja” OR Bonda OR Remo OR Chakhesang OR Chang OR Chenchu OR Chenchucoolam OR Chenchwar OR Chenswar OR Choncharu OR Chote OR Chowte OR Chawtes OR Purum OR Dal OR “Dandami Marias” OR “Bison Horn Marias” OR “Kalpati Marias” OR “Singh Marias” OR “TalaguDDa Marias” OR Maria OR Dhodia OR Dhobi OR Dhori OR Dhore OR Dhowari OR Doria OR Didayi OR Gataq OR Getaq OR Geta' OR Gta' OR “Gta Asa” OR Didei OR Dire OR Gata' OR Didayee OR Digaro-Mishmi OR Digaru-Mishmi OR Taraon OR “Dimasa Kachari” OR Dimasa OR Dima-fisa OR Dogra OR Dogri OR Dogri-Kangri OR Dhogaryali OR Dogari OR “Dogri Jammu” OR “Dogri Pahari” OR Dogri-Kangr OR Gaddis OR Gaddies OR Garo OR Achik OR Abeng OR Ambeng OR Awe OR Ruga OR Atong OR Garrow OR Mande OR Gowlan OR Gujjars OR Halbaa OR Halba OR Halbi OR Hmar OR Mhar OR Mar OR Ho OR Lanka Kol OR “Bihar Ho” OR “Idu Mishmi” OR “Yidu Lobha” OR Chulikatas OR Irula OR Jaintias OR Jayantias OR Syntengs OR Pnars OR Hynniewtrep OR Jarawa OR “Jenu Kurumba” OR “Jennu Kurumba” OR “Jenu Kuruba” OR “Kadu Nayikas” OR Juang OR Patuas OR Puttooas OR Patra-Saara OR Patta-Savara OR Juango OR Kabui OR Rongmei OR Zeliangrong OR Puimei OR Inpui OR Kapwi OR Koboi OR Kubai OR “Kabui Naga” OR “Kacha Naga” OR “Kadu Kuruba” OR “Kadu Kurumba” OR Khasi OR Khoibu OR “Khoibu Maring” OR “Khoibu Maring Naga” OR Khond OR Kandhs OR “Raj Khonds” OR Kinnaure OR Kinners OR Kinnauris OR Kisan OR Nagasia OR Nagesia OR Nagesar OR Naksia OR Diharia OR Oraon OR Dhangad OR Dhangar OR Dhanka OR Kuda OR Kurukh OR Kurunkh OR Orao OR Uraon OR Kondh OR Kond OR Kui OR Buda Kondh OR “Bura Kandha” OR “Desia Kandha” OR “Dungaria Kondh” OR “Kutia Kandha” OR “Kandha Gauda” OR “Muli Kondh” OR “Malua Kondh” OR “Pengo Kandha” OR “Raja Kondh” OR “Raj Khond” OR “Desia Kondh” OR “Dongariya Kondh” OR Korku OR Bondhi OR Bopchi OR Kodaku OR Kurku OR Mouasi OR Muwasi OR Koya OR Koi OR “Koi Gondi” OR Kavor OR Koa OR Koitar OR Koyato OR Kaya OR Koyi OR Raj Koya OR Kavor OR Koitor OR Koithur OR Koitur OR Kutchi OR Kacchi OR Kanbis OR Bhanushali OR Rabari OR Ahirs OR Meghwals OR Lahules OR Lahulas OR Lahaulis OR Liangmai OR Kacha OR Liyang OR Lyengmai OR Liangmei OR Lyangmay OR Lohara OR Lohra OR Luhura OR Luhara OR Lotha OR “Naga Lotha” OR Madia OR Madia-Gond OR Maria OR Maria-Gond OR Madiya OR “Hill Madia” OR “Bison Horn Maria” OR Magahi OR Magadhi OR Magaya OR Maghai OR Maghaya OR Maghori OR Magi OR Magodhi OR Bihari OR Megahi OR “Magar Eastern” OR Magari OR Mangar OR Mangari OR Magarkura OR Mahali OR Mahli OR Mao OR “Naga Mao” OR Maram OR “Naga Maram” OR Meithei OR Meitei OR Manipuris OR Kathi OR Kathe OR Ponna OR Meiteilon OR Miju-Mishmi OR Kaman OR Mishing OR Mising OR Takam OR Tanis OR Amis OR Monpa OR Mendba OR Moinba OR Monba OR Menpa OR Mongba OR Menba OR Monsang OR Moshang OR Monshang OR Mushang OR Mawshang OR Munda OR Colh OR Hor OR Kaur OR Mudus OR Mura OR Haroko OR Horohon OR Manki OR Mundu OR “Nicobarese Southern” OR Nicobara OR Nishi OR Nissi OR Dafla OR Nishang OR Nishing OR Nocte OR Bordari OR Panidori OR Namsangia OR Onge OR Ong OR Oraon OR Uraon OR Khurukh OR Paite OR Chin OR Kuki OR Lushai OR Tedim OR Sahte OR Zou OR Paliyan OR Paliyar OR Palleyan OR Palliyar OR Pangwali OR Pahari OR Pangi OR “Pangwali Pahari” OR Pangwala OR Piral OR Pochury OR Poumai OR Raika OR Rabari OR Rebari OR Rabha OR Rahbari OR Maru OR Godwar OR Pitalia OR Chalkia OR Rabbari OR Sorthia OR “Sorathi Charalia” OR Charmta OR Luni OR Kushar OR Tank OR “Muchhal Ka” OR Dhebariya OR Dheberya OR Vagadiya OR Vagariya OR Desi OR Kutchi OR Bhopa OR Gujarati OR Mogha OR Vishotar OR Sinai OR Rengma OR Sangtam OR Santali OR Hor OR Har OR Satar OR Santhali OR Santhal OR Sandal OR Sangtal OR Santal OR Sentali OR Samtali OR Santhiali OR Sonthal OR Saora OR Sora OR Saora OR Saonras OR Shabari OR Sabar OR Saura OR Savara OR Sawaria OR Swara OR Sabara OR Savara OR Sema OR Simi OR Sumi OR “Naga Sumi” OR Sentinel OR Sentinelese OR “Shom Peng” OR “Shom Pen” OR Shompeng OR Shompen OR Shobang OR Kalay OR Keyet OR Spitians OR Sulung OR Sullung OR Suling OR Sulong OR Puroik OR Pariok OR Tagin OR Tani OR “Apa Tani” OR Tangkhul OR Tagkhul OR Thangkhulm OR Champhung OR Luhuppa OR Luppa OR Somra OR Hao OR Tutsa OR Totcha OR Vaiphei OR Bhaipei OR Vaipei OR Veiphei OR Wancho OR “Banpara Naga” OR Joboka OR Warli OR Varli OR Yerukula OR Yerukala OR Yarukula OR Yerkula OR Yerukla OR Erukala OR Korava OR Yerukala-Korava OR Yerukula-Bhasha OR “Eruku Bhasha” OR Korchi OR Kurutha OR “Kurru Bhasha” OR Zangskari OR Zanskari OR Zaskari OR Zomi OR Zo |

| MALDIVES: SEAR D |
| --- |
| Indigenous OR aborigin* OR native OR “first nation*” OR “ethnic group” OR tribal OR tribe OR autochthonous |

| MYANMAR OR BURMA: SEAR D |
| --- |
| Indigenous OR aborigin* OR native OR “first nation*” OR “ethnic group” OR tribal OR tribe OR autochthonous OR Shan OR Karen OR Rakhine OR Mon OR Chin OR Kachin OR Karenni OR Akha OR Arakanese OR Maghi OR Marma OR Mogh OR Rakhine OR Chin OR Danau OR Danaw OR Danu OR Kachin OR Chingpaw OR Singphos OR Karen OR Pgaganyaw OR Plong OR Pwo OR Sgaw OR Skaw OR S'waw OR Karenni OR Kayah OR Kayan OR Kayaw OR Padaung OR Paku OR Kokang OR Kuki OR Mon OR Nagas OR Rohingya OR Shan OR Tavoyan OR Wa OR Hkawa OR Kala OR Kawa OR Lawa OR Va |

| NEPAL: SEAR D |
| --- |
| Indigenous OR aborigin* OR native OR “first nation*” OR “ethnic group” OR tribal OR tribe OR autochthonous OR “Adivasi Janajati” OR Angika OR Anga OR Angikar OR Chhika-Chhiki OR Awadhi OR “Awadhi Abadhi” OR Bahing OR “Bahing Rai” OR Bantawa OR “Bantawa Rai” OR Baraamu OR Baram OR Baramu OR Brahmu OR Bramu OR Bhramu OR Barhamu OR Bhojpuri OR Chukwa OR “Cukwa Ring” OR Pohing OR “Pohing Kha” OR Darai OR Darwai OR Dahri OR Daree OR Daroe OR Darmiya OR Darimiya OR Darmani OR Sauka OR Shauka OR Dhimal OR Haiko OR “Limbus of Terai” OR Dzongkha OR Jonkha OR “Bhotia of Bhutan” OR Zongkhar OR Drukke OR Drukha OR Bhutanese OR “Helambu Sherpa” OR “Yolmo Sherpa” OR Hyolmo OR Jerung OR Jero OR Jirel OR Jiripas OR Jiripa OR Jirpa OR Jiri OR Jirial OR Zaral OR Ziral OR Kagate OR Bhotia OR “Kagate Bhote” OR “Kagate Bhotia” OR Kagatey OR Kagati OR Limbu OR Yakthung OR Magar OR Western OR Mangar OR Maithili OR Maitili OR Maithil OR Majhi OR Bhumar OR Manangba OR “Manang Bas” OR Nyishangba OR Nyi-Shang OR Manang OR Manangi OR Manangpa OR Manangbolt OR Neshyang OR Nesyangba OR Nyeshang OR Mugali OR Mugom OR Mugu OR Kham OR Khan OR Mugum OR Tamang OR Mustang OR Lo OR Lowa OR Mastang OR Sherpa OR Sharpa OR “Sharpa Bhotia” OR Xiaerba OR Serwa OR Tamang OR Thudam OR “Thudam Bhote” OR Thudambas OR Bhote OR Thulung OR “Thulunge Rai” OR “Thulu Luwa” OR Thululoa OR “Thulung La” OR “Thulung Lo” OR “Thulung Jemu” OR “Toaku Lwa” OR “Sub-Group of the Rai” |

| AUSTRALIA: WPR A |
| --- |
| Indigenous OR aborigin* OR native OR “first nation*” OR “ethnic group” OR tribal OR tribe OR autochthonous OR “Torres Strait*” |

| BRUNEI: WPR A |
| --- |
| Indigenous OR aborigin* OR native OR “first nation*” OR “ethnic group” OR tribal OR tribe OR autochthonous OR Dusun OR Bisaya OR Murut OR Kedayan OR Iban OR Tutong OR Penan |

| JAPAN: WPR A |
| --- |
| Indigenous OR aborigin* OR native OR “first nation*” OR “ethnic group” OR tribal OR tribe OR autochthonous OR Ryūkyūans OR Okinawans OR Ainu OR Utari |

| “NEW ZEALAND”: WPR A |
| --- |
| Indigenous OR aborigin* OR native OR “first nation*” OR “ethnic group” OR tribal OR tribe OR autochthonous OR Māori |

| CAMBODIA: WPR B |
| --- |
| Indigenous OR aborigin* OR native OR “first nation*” OR “ethnic group” OR tribal OR tribe OR autochthonous OR “Khmer Loeu” OR Kachac OR Chamic OR Kachak OR Kreung OR Krung OR Kru'ng OR Tampuon OR Campuon OR “Kha Tampuon” OR Proon* OR Tamphuan OR Tampuen OR Tumpun |

| CHINA: WPR A |
| --- |
| Indigenous OR aborigin* OR native OR “first nation*” OR “ethnic group” OR tribal OR tribe OR autochthonous OR “national minority” or Zhuang OR Manchu OR Hui OR Uyghur OR Yi OR Lolo OR “Tujia Achang” OR Bai OR Blang OR Bulang OR Wa OR Samtuan OR Samtao OR Saamtaav OR Pulang OR Bonan OR Bouyei OR Bouyi OR Buyei OR Buyi OR Dai OR Tai OR Baijue OR “Lu Dai” OR Daur OR De'ang OR Dong OR Gam OR Kam OR Tong OR Tung OR Dongxiang OR Tunghsiang OR Santa OR “Mongolian Huihui” OR Drung OR Ewenki OR Evenki OR Ewenke OR “Manchurian Solon” OR “Owenke Solon” OR “Solon Evenki” OR Suolun OR Tungus OR Gelo OR Hani OR Akha OR Biyo OR Bio OR Biyue OR Kado OR Mahei OR Pudu OR Putu OR Sansu OR Hezhen OR Hezhe OR Sushen OR Hmong OR Jing OR Jingpo OR Jino OR Kazak OR Kazakh OR Khmu OR Kirjiz OR Lahu OR Lhoba OR Li OR Lisu OR Maonan OR Miao OR “Black Hmong” OR “Black Miao” OR Daishou OR Guoxiong OR “Long Skirt Miao” OR Mao OR “Red Hmong” OR “Red Miao” OR “Short Skirt Miao” OR “White Hmong” OR Moinba OR Mosuo OR Moso OR Musuo OR Mulam OR Naxi OR Nahsi OR Nasi OR Nakhi OR Lomi OR Mu OR Nisu OR Nu OR Oroqen OR Ozbek OR Pumi OR Qiang OR Salar OR She OR Shui OR Shuijia OR Sui OR “Sui Li” OR Suijia OR Suipo OR Tajik OR Tartar OR Tatar OR Tata’er OR Dada OR Daden OR Tu OR Tujia OR Uygur OR Va OR Xibe OR Yao OR “Baiku Yao” OR Baikuyao OR Bingduoyou OR Bunu OR “Guoshan Yan” OR Guoshanyao OR “Hon Yao” OR Jinmen OR Lajia OR Mian OR “Pan Yao” OR Panyao OR “Pindi Yao” OR Pindiyao OR “Shanzi Yao” OR Shanziyao OR Ajia OR “Black Yi” OR Yi OR Heiyi OR Qunuo OR Wajia OR “White Yi” OR Xiaxi OR Younuo OR “Hong Yao” OR “Red Yao” OR Yunou OR Yuno OR Yugur |

| “COOK ISLANDS”: WPR B |
| --- |
| Indigenous OR aborigin* OR native OR “first nation*” OR “ethnic group” OR tribal OR tribe OR autochthonous |

| FIJI: WPR B |
| --- |
| Indigenous OR aborigin* OR native OR “first nation*” OR “ethnic group” OR tribal OR tribe OR autochthonous OR Rotumans OR iTaukei |

| KIRIBATI: WPR B |
| --- |
| Indigenous OR aborigin* OR native OR “first nation*” OR “ethnic group” OR tribal OR tribe OR autochthonous OR I-Kiribati OR Tuvalu OR Banaba |

| LAO*: WPR B |
| --- |
| Indigenous OR aborigin* OR native OR “first nation*” OR “ethnic group” OR tribal OR tribe OR autochthonous OR Phouthay OR Tai OR Makong OR Katang OR Lue OR Akha OR Aka OR Ekaw OR Ekwa OR Kaw OR Khka OR “Kon Ak'a” OR Hmong OR Maio OR Meo OR Khmu OR Kammu OR Khamu OR Lamet OR “Kha Lamet” OR Khamet OR Khamed OR Lemet OR Rmeet OR Lantan OR Lantien OR Malabri OR Mlabri OR “Phi Tong Luang” OR “Toong Luang” OR “Yellow Leaf” OR Yumbr OR Yumbri |

| MALAYSIA: WPR B |
| --- |
| Indigenous OR aborigin* OR native OR “first nation*” OR “ethnic group” OR tribal OR tribe OR autochthonous OR “Anak Negeri” OR “Orang Ulu” OR Dayak OR “Orang Asli” OR Bajau OR Illanun OR Badjao OR Badjau OR Bajaw OR Bajo OR Suluk OR Obian OR “Orang Sama” OR “Sama Dilaut” OR “Sea Gypsies” OR Binadan OR Batek OR “Batek Negritos” OR Bidayuh OR “Bukar Sadong” OR Tebakang OR Bugis OR Buginese OR Luwu OR Ugi OR Chewong OR “Che’Wong” OR Iban OR “Sea Dayak” OR Jahai OR Jah OR Jehai OR Pangan OR “Jah Hut” OR Cheres OR Jakun OR Djakun OR “Orang Hulu” OR “Kadazan Dusun” OR “Tuaran Dusun” OR “Suang Lotud” OR Minokok OR Ringus OR “Tempasuk Dusun” OR Tindal OR “Orang Sungai” OR Kedayan OR Kedyan OR Kadayan OR Kadien OR Kensiu OR Negrito OR Lanoh OR Sakai OR Semnan OR “Mah Meri” OR Besisi OR Btsisek OR Melanaus OR Balingian OR Belanau OR Bruit OR Dalat OR Sarikei OR Muka OR Melanau OR Melenau OR Mendriq OR Mendrik OR Menri OR Mandriq OR Murut OR Timogun OR Tagal OR Nabas OR Penan OR Semai OR “Semai Senoi” OR Semang OR Semelai OR “Semaq Tasik” OR “Semoq Beri” OR Semaq OR Semalai OR “Semaq Beri” OR “Jakun of Tekai River” OR Senoi OR Sengoi OR Temiar OR “Temiar Senoi” OR Bumiputera OR Temuan OR Belanda OR Biduanda OR Benua OR Mantera OR Kenyah OR Kayan OR Lunbawang OR Punan OR Bisayah OR Kelabit OR Berawan OR Kejaman OR Ukit OR Sekapan OR Paitan |

| “MARSHALL ISLANDS”: WPR B |
| --- |
| Indigenous OR aborigin* OR native OR “first nation*” OR “ethnic group” OR tribal OR tribe OR autochthonous |

| MICRONESIA: WPR B |
| --- |
| Indigenous OR aborigin* OR native OR “first nation*” OR “ethnic group” OR tribal OR tribe OR autochthonous |

| MONGOLIA: WPR B |
| --- |
| Indigenous OR aborigin* OR native OR “first nation*” OR “ethnic group” OR tribal OR tribe OR autochthonous OR Bayad OR Bayaad OR Bayit OR Bait OR Dariganga OR Durvud OR Durbet OR Dörbed OR Dörvöd OR Kazakh OR Kazak OR Qazaq OR Khalkha OR Halh OR Mingat OR Myangad OR Torguud OR Torgut OR Tsaatan OR Dukha OR Tsachin |

| NAURU: WPR B |
| --- |
| Indigenous OR aborigin* OR native OR “first nation*” OR “ethnic group” OR tribal OR tribe OR autochthonous |

| NIUE: WPR B |
| --- |
| Indigenous OR aborigin* OR native OR “first nation*” OR “ethnic group” OR tribal OR tribe OR autochthonous |

| PALAU: WPR B |
| --- |
| Indigenous OR aborigin* OR native OR “first nation*” OR “ethnic group” OR tribal OR tribe OR autochthonous |

| “PAPUA NEW GUINEA”: WPR B |
| --- |
| Indigenous OR aborigin* OR native OR “first nation*” OR “ethnic group” OR tribal OR tribe OR autochthonous OR Bougainvilleans |

| PHILIPPINES: WPR B |
| --- |
| Indigenous OR aborigin* OR native OR “first nation*” OR “ethnic group” OR tribal OR tribe OR autochthonous OR Tagalog OR Bisaya OR Binisaya OR Cebuano OR Ilocano OR Hiligaynon OR Ilonggo OR Bikol OR Bicol OR Waray OR Igorot OR Lumad OR Mangyan OR Abaknon OR Capul OR “Capul Samal” OR Capuleno OR Inabaknon OR Inbaknon OR Kapul OR Sama OR Applai OR Appais OR Kankanan-ey OR Katangnan OR “Lepanto Igorot” OR “Sagada Igorot” OR “Western Bontoc” OR “Western Bontok” OR Arumanen OR Aromanon OR Arumamen OR “Central Mindanao” OR Ilianen OR Liringanen OR Manobo OR Manuvu OR “South Cotabato” OR Attaw OR Bagobo OR Clata OR Diangan OR Giangan OR Guingan OR Guiangan OR Gulanga OR Jangan OR Klata OR Obo OR Banwaon OR Adgawanon OR Banuaonon OR Banwanon OR Higaonon-Banwaon OR “Bontok Igorots” OR Bontoc OR Kadaklan-Barlig OR Bukidnon OR Binokid OR Binukid OR “Central Bukidnon” OR Butuanon OR Lapaknon OR “Davao Chabakano” OR Chabakano OR “Chabakano Creole” OR Chavacano OR Creole OR Davao OR Zamboanga OR Dibabawon OR Dibabaon OR Mandaya OR “Dibabawon Manobo” OR “Digagaon Mandaya Manobo” OR “Orang Dibabawon” OR Higaonon OR Banuanon OR Higanon OR “Higaonon Manobo” OR “Misamis Higaonon” OR Talaandig OR “Jama Mapun” OR “Orang Cagayan” OR “Tao Cagayan” OR Kabihug OR Abian OR Aeta OR Agiyan OR Agta OR Bihug OR Bikol OR “Camarines Norte Agta” OR Manide OR Negrito* OR Lambangian OR Teduray-Lambangian OR Tiruray OR Lapuyan OR Lapuyen OR “Subanun Lapuyan” OR Margosatubig OR Subanon OR Subanun OR Subanen OR Suban-on OR “Southern Subanun” OR “Manobo Agusan” OR “Agusan del Sur” OR Agusan OR Higanon OR Kidapawan OR Ubo OR Molbog OR Molebugan OR Molebuganon OR Molebuganori OR Palawan OR Palawano OR Palawanon OR Pala’wan OR Pinalawan OR Sangil OR Sanggil OR Sangire OR Sangihe OR “Sangir Pilipinas” OR Sangir OR Sangu OR Marore OR Sangirezen OR Talaoerezen OR Surigaonon OR Surigao OR Tagakaolo OR Kalagan OR Mansaka OR “Tagakaolo Kalagan” OR Tagakaulu OR “Tagakaulu Kalagan” OR Tagbanua OR Tagbanuas OR Tala-Andig OR Talandig OR “Tau´t Batu” OR “Tao’t Bato” OR “Tao’t Batu” OR “Taw Batu” OR TBoli OR Kiamba OR Tagabeli OR Tagabulu OR T'boli OR Tibole OR Tiboli |

| “REPUBLIC OF KOREA”: WPR B |
| --- |
| Indigenous OR aborigin* OR native OR “first nation*” OR “ethnic group” OR tribal OR tribe OR autochthonous |

| SAOMA: WPR B |
| --- |
| Indigenous OR aborigin* OR native OR “first nation*” OR “ethnic group” OR tribal OR tribe OR autochthonous |

| “SOLOMON ISLANDS”: WPR B |
| --- |
| Indigenous OR aborigin* OR native OR “first nation*” OR “ethnic group” OR tribal OR tribe OR autochthonous |

| TONGA: WPR B |
| --- |
| Indigenous OR aborigin* OR native OR “first nation*” OR “ethnic group” OR tribal OR tribe OR autochthonous |

| TUVALU: WPR B |
| --- |
| Indigenous OR aborigin* OR native OR “first nation*” OR “ethnic group” OR tribal OR tribe OR autochthonous |

| VANUATU: WPR B |
| --- |
| Indigenous OR aborigin* OR native OR “first nation*” OR “ethnic group” OR tribal OR tribe OR autochthonous OR Wallisians OR Futunans OR i-Kiribati |

| VIETNAM: WPR B |
| --- |
| Indigenous OR aborigin* OR native OR “first nation*” OR “ethnic group” OR tribal OR tribe OR autochthonous OR Akha OR Aka OR Ak’a OR Ahka OR Hani OR “Ha Nhi” OR Ikaw OR Xo OR Khako OR “Kha ko” OR “Khao Ikor” OR Aini OR Yani OR “Hka Ko” OR “Khao Kha Ko” OR Arem OR A-Rem OR Chomrau OR Chombrau OR Umo OR Bahnar OR “Ba Na” OR “To Lo” OR Golar OR Jolong OR “Gio Lang”OR “Y Lang” OR “Ro Ngao” OR Reungao OR Rangao OR Ro-Ngao OR “Bahnar Rongao” OR Krum OR Krem OR Roh OR “Con Kde” OR “Kpang Cong” OR “Bo Mon” OR Bonom OR Bomom OR Alacong OR Alakong OR “A-La Cong” OR Brao OR Brau OR Braou OR Proue OR Proon OR Brou OR “Cao Lan” OR Caolan OR “Hon Ban” OR “San Chay” OR “San Chi Man Cao-Lan” OR Sán-Chi OR Mán OR “Cao Lan-Sán Chi” OR “Cho Ro” OR “Chau Ro” OR Chauro OR Chorao OR Choro OR Cho-ro OR Chrau OR “Do Ro” OR Zro OR “Chu Ru” OR Cadoe OR Chru OR Choru OR “Cho Ru” OR Chu OR Chu-ru OR Churu OR Cru OR Degar OR Kru OR Loang OR Seyu OR Ru OR Ede OR E-de OR Edeh OR De OR Dega OR Haqniq OR “Ha Nhi” OR Hanízú OR Hanhi OR “H Nhi” OR “Ha Nhi Gia” OR Uni OR ”U Ni” OR Xauni OR ”Xa U Ni” OR Koho OR Coho OR Co-ho OR Ko-ho OR Kohor OR K'ho OR Caho OR “Co Ho” OR “La Ha” OR “Xa Khan” OR “Xa Cah” OR “Xa Chien” OR “Xa Khao” OR “Xa Lay” OR “Xa Lga” OR “Khla Don” OR “Kla Dong” OR “Khla Liik” OR “La Hu” OR Luohei OR Launa OR Lahuna OR Laku OR Kaixien OR Namen OR Mussuh OR Muhso OR Musso OR Mussar OR Mussur OR Moso OR Lachi OR Lati OR “Cu Te” OR “Tho Den” OR “Black Tho” OR “Man La” OR Chi OR “La Chi” OR Pula OR Phula OR Fula OR Foula OR Lipupo OR Laji OR Lipulio OR Laqua OR “Y Pi” OR “Y Pong” OR Laghuu OR Laopa OR Xapho OR “Xa Pho” OR “Lahu Shi” OR “Yellow Lahu” OR Kouy OR Lu OR Duon OR Kon OR Leu OR “Lu Ge Zi” OR “Lu Ren” OR Lue OR Lugepo OR Nhuon OR Zhon OR Maa OR Ma OR “Chau Ma” OR “Ma Xop” OR “Ma To” OR “Ma Krung” OR “Ma Ngan” OR Maleng OR Pakatan OR Malieng OR Malang OR “Ma Leng” OR “Ma Lieng” OR Romam OR “Ro Mam” OR Ro-mam OR Sedang OR Hadang OR Hdang OR Hoteang OR Roteang OR Rotea OR Hotea OR “Xo Dang” OR Xodangg OR “Xa Dang” OR Cadong OR Tang OR Kmrang OR Kmrong OR Konelane OR Brila OR Stieng OR Budeh OR Xtieng OR “Xa Dieng” OR “Ba Ra” OR “Bu Dip” OR Budip OR “Bu Lanh” OR Rangah OR Tay OR Tho OR Ngan OR Phen OR “Thu Lao” OR “Pa Di” |

Each search will comprise: A. country AND

B. parasites/bacteria terms AND

C. country relevant indigenous terms

**Appendix 3:** Example search strategy for Indonesia.

Indonesia **AND** “soil transmitted helminth*” OR STH OR Ascaris OR Trichuris OR Nectator OR Ancylostoma OR hookworm* OR Strongyloides OR malaria* OR plasmodi* OR

tuberculosis OR TB OR “Mycobacterium tuberculosis” **AND** Indigenous OR aborigin* OR native OR first nation* OR “ethnic group” OR tribal OR tribe OR autochthonous OR “adat terpencil” OR Acehnese OR Achinese OR Atjeher OR “Orang Aceh” OR Acehnais OR Acehno OR Atjeh OR Atjehnese OR Achehnese OR Achenese OR Adabe OR Ataura OR Atauru OR Atauro OR Raklu-Un OR “Raklu Un” OR Adonara OR “Tusa Tadon” OR Waiwerang OR Vaiverang OR Sagu OR Alorese OR Ampanang OR Andio OR Masama OR Andio'o OR Imbao'o OR Aralle OR Tabulahan OR Asmat OR Asamat OR Asemer OR Asomat OR Bagusa OR “Batak Alas-Kluet” OR “Alas-Kluet Batak” OR “Batak Kluet-Alas” OR “Kluet-Alas Batak” OR “Alas Kluet” OR “Kluet Alas” OR Alas OR Kluet OR “Batak Angkola” OR “Orang Angkola” OR Anakola OR Angkola OR “Batak Dairi” OR Dairi OR “Dairi Batak” OR “Orang Batak Dairi” OR Pakpak OR “Pakpak Dairi” OR Sumut OR “Batak Karo” OR “Karo Batak” OR “Orang Batak Karo” OR Karonese OR “Batak Mandailing” OR “Mandailing Batak” OR Batta OR “Orang Mandailing” OR “Batak Simalungun” OR “Simalungun Batak” OR “Orang Batak Simalungun” OR Simelungun OR Simelungan OR Timur OR “Batak Toba” OR “Toba Batak” OR “Orang Batak Toba” OR “Silindung Batak” OR Bauzi OR Baudi OR Bauri OR Baudji OR Baudzi OR Damal OR Uhunduni OR Amung OR “Amung Kal” OR Amungme OR Amuy OR Enggipiloe OR Hamung OR Oehoendoeni OR Dani OR Gayo OR “Orang Gayo” OR Gayonese OR Ketengban OR Kupel OR Oktengban OR Kombai OR Komboy OR Kubu OR Djambi OR “Orang Darat” OR Mentawai OR Mentawei OR Mentawi OR Minangkabau OR Minang OR Padang OR “Orang Minangkabau” OR Moni OR Migani OR Djonggunu OR Jonggunu OR Moronene OR Maronene OR Nias OR Batu OR Nuaulu OR “Southern Nuaulu” OR “Northern Nuaulu” OR Rejang OR “Keme Tun Djang” OR “Orang Rejang” OR Djang OR “Tun Djang” OR “Redjang Empat Petulai” OR “Djang Lebong” OR “Djang Bele Tebo” OR “Djang Musai” OR “Djang Lai” OR “Djang Bekulau” OR “Djang Abeus” OR “Djang Aweus” OR “Bang Hadji” OR Semitul OR Sawang OR Selako OR “Selako Dayak” OR Selakau OR Salakau OR Salako OR Silakau OR Tamiang OR Malayu OR Wandamen OR Wandamen-Windesi OR Windesi OR Windessi OR Bintuni OR Bentuni OR Bentoeni OR Wamesa OR Wolio OR Buton OR Butonese OR Walio

**Appendix 4:** Data extraction tool.

The following headings will be used for data extraction within Excel (version 2014):

- First author
- Year of publication
- Year of study/data collection
- Study design
- Country
- Village, region/state
- Population group(s) (minority indigenous/other )
- Name of population group(s) (e.g., Aeta, Bulang, Penan)
- Study site (e.g., school, community etc)
- Sample type(s) (e.g., blood, fecal)
- Number of samples taken and analyzed per participant
- Infectious agent(s) (e.g., A.lumbricoides, P.falciparum)
- Diagnostic method(s) (e.g., smear microscopy, culture, chest X-ray, and GenXeprt for active TB; microscopy, RDT, PCR, splenomegaly for malaria and microscopy, PCR, serology, for STH )
- Study population (children, adult, both)
- Study population age group (<15 years; ≥15 years)
- Study population median age
- Study population size (n)
- Male (# male within the study population)
- Female (# female within the study population)
- Number of people infected
- Co-infection (name of infectious agent)
- Prevalence of co-infection (# co-infected)
- Comments/notes

**Appendix 5:** Quality and bias assessment.

The following adaption to the Newcastle-Ottawa Scale ^9^ will be utilized for this review:

| **Newcastle-Ottawa Scale adapted for cross-sectional studies (Maximum total= 9 points)** | |
| --- | --- |
|  | |
| **Study Population** | |
| 1 | The study population is clearly defined |
| 0 | The study population is not clearly defined |
| **Representativeness of the sample** | |
| 2 | Study sample is representative of the study population (all subjects or random sampling) |
| 1 | Study sample comprises a select group of the study population (non-random sampling) |
| 0 | No description of the sampling strategy. |
| **Ascertainment of specimen collection methods** | |
| 1 | The study clearly defines specimen collection methodologies |
| 0 | The study does not detail specimen collection methodologies |
| **Sample size** | |
| 1 | Justified and satisfactory (sample size and power calculation included) |
| 0 | Not justified |
| **Non-respondents** | |
| 1 | Comparability between respondents and non-respondent’s characteristics are established |
| 0 | No description of the response rate or the characteristics of the responders and the non-responders. |
| **Comparability:** | |
| **Impact of Bias (selection bias, measurement bias, participant reporting, confounders)** | |
| 1 | Where relevant, the study acknowledges and mitigates for potential bias. When comparisons are made between different study populations results are adjusted for confounders |
| 0 | Where appropriate, the study does not acknowledge or mitigate for potential bias. When comparisons are made between different study populations results are not adjusted for confounders |
| **Assessment of the outcome (TB, Malaria, STH infection)** | |
| 1 | Objective diagnostic methodology with units of measurement and /or definitions |
| 0 | No definitive diagnosis or self-report |
| **Statistical analysis** | |
| 1 | The statistical method used is clearly described and appropriate for the analysis undertaken. Where comparisons are made between population groups, the measurement of the association is presented, including confidence intervals and the probability level (p value) |
| 0 | The statistical method is inappropriate/not described/incomplete |

**References**

1. BMC Systematic Reviews. Preparing your manuscript: Protocol [Internet]. [cited 10.08.20]. Available from: <https://systematicreviewsjournal.biomedcentral.com/submission-guidelines/preparing-your-manuscript/protocol>

2. Moher D, Shamseer L, Clarke M, Ghersi D, Liberati A, Petticrew M, et al. Preferred reporting items for systematic review and meta-analysis protocols (PRISMA-P) 2015 statement. Systematic reviews. 2015;4(1):1.

3. Moher D, Stewart L, Shekelle P. Implementing PRISMA-P: recommendations for prospective authors Springer; 2016.

4. World Health Organization. Global Burden of Disease Regions used for WHO-CHOICE Analyses [Internet]. n.d. [cited 05.07.19]. Available from: <https://www.who.int/choice/demography/regions/en/>

5. World Health Organization. List of Member States by WHO Region and Mortality Stratum [Internet]. [cited 10.06.20]. Available from: <https://www.who.int/choice/demography/mortality_strata/en/>

6. Minority Rights Group International. World Directory of Minorities and Indigenous Peoples [Internet]. n.d. [cited 29.06.19]. Available from: <https://minorityrights.org/directory/>

7. Native Planet. Indigenous Mapping- Ethnic Minority Groups from Asia [Internet]. n.d. [cited 02.07.19]. Available from: <https://www.nativeplanet.org/indigenous/ethnicdiversity/indigenous_data_asia.shtml>

8. Network AIPPFaIKaP. Who We Are Indigenous Peoples in Asia [Internet]. 2009 [Available from: <https://www.iwgia.org/images/publications/0640_ho_are_e_IPs_in_Asia.pdf>

9. Wells GA, Shea B, O'Connell D, Peterson J, EWelch V, Losos M, et al. The Newcastle-Ottawa Scale (NOS) for assessing the quality of nonrandomised studies in meta-analyses [Internet]. 2019 [cited 06.05.20]. Available from: <http://www.ohri.ca/programs/clinical_epidemiology/oxford.asp>
